# An Easy-To-Use Simulation Program Demonstrates Variations in Bacterial Cell Cycle Parameters Depending on Medium and Temperature

**DOI:** 10.1371/journal.pone.0030981

**Published:** 2012-02-13

**Authors:** Caroline Stokke, Ingvild Flåtten, Kirsten Skarstad

**Affiliations:** Department of Cell Biology, Institute for Cancer Research, The Norwegian Radium Hospital, Oslo University Hospital, Oslo, Norway; Texas A&M University, United States of America

## Abstract

Many studies are performed on chromosome replication and segregation in *Escherichia coli* and other bacteria capable of complex replication with C phases spanning several generations. For such investigations an understanding of the replication patterns, including copy numbers of origins and replication forks, is crucial for correct interpretation of the results.

Flow cytometry is an important tool for generation of experimental DNA distributions of cell populations. Here, a Visual Basic based simulation program was written for the computation of theoretical DNA distributions for different choices of cell cycle parameters (C and D phase durations, doubling time etc). These cell cycle parameters can be iterated until the best fit between the experimental and theoretical DNA histograms is obtained. The Excel file containing the simulation software is attached as supporting information.

Cultures of *Escherichia coli* were grown at twelve different media and temperature conditions, with following measurements by flow cytometry and simulation of the DNA distributions. A good fit was found for each growth condition by use of our simulation program. The resulting cell cycle parameters displayed clear inter-media differences in replication patterns, but indicated a high degree of temperature independence for each medium. The exception was the poorest medium (acetate), where the cells grew with overlapping replication cycles at 42°C, but without at the lower temperatures.

We have developed an easy-to-use tool for determination of bacteria's cell cycle parameters, and consequently the cells' chromosome configurations. The procedure only requires DNA distribution measurements by flow cytometry. Use of this simulation program for *E. coli* cultures shows that even cells growing quite slowly can have overlapping replication cycles. It is therefore always important not only to assume cells' replication patterns, but to actually determine the cell cycle parameters when changing growth conditions.

## Introduction

Unlike eukaryotic cells, where many origins on many chromosomes initiate throughout the replication period [1], [2], most bacteria contain one circular chromosome with a single origin. The bacterial cell cycle is divided into three parts; a period from cell “birth” to initiation of replication (B phase, equivalent to the eukaryotic G1 phase), a period required for replication (C phase, equivalent to S phase) and the time between termination of replication and cell division (D phase, equivalent to G2/M phase). In order to grow with shorter doubling times than the combined time required for replication and segregation (C+D) several types of bacteria have the ability to initiate replication in preceding generations [3], [4]. Initiation of replication then occurs at two origins in the “mother” cell, or even at four origins in the “grandmother” cell.

Our developed simulation program was utilized for analyses of *Escherichia coli*, the most widely studied of bacteria. The *E. coli* chromosome is replicated bidirectionally from the origin, and in rapidly growing cells all copies of the origin will initiate replication at the same cell age [5]. After the discovery that *E. coli* was capable of initiating C phase in previous generations (displaying “multi-fork replication”), it was suggested that C and D phase durations were constant, approximately 40 and 20 minutes respectively for B/r strains during rapid growth [3]. These periods have later been shown to vary with growth conditions and nutrient availability [6]. Different strains of *E. coli* grown in the same media have also displayed diverse C and D phase durations [7], [8], [9].

Flow cytometry has proven very useful for cell cycle analysis by the generation of DNA content histograms containing information from thousands of bacterial cells in just minutes [10]. Previous simulation programs have also been based on the fitting of theoretical to experimental DNA histograms, but of these some are written in out-dated versions of computing languages [11], some are only valid for cells without multi-fork replication [12] and some have other limitations and can therefore not be used for all growth conditions [13]. In this work we have developed a new program for simulation of DNA distributions for all one-chromosome bacterial cells grown with or without overlapping replication rounds. Use of this software only requires knowledge of Excel because the simulation code is provided as a separate Visual Basic macro.

## Methods

### Growth conditions

The bacterium used was *Escherichia coli* K-12 strain MG1655 [14]. Cells were grown in four different media at 30°C, 37°C and 42°C, i.e. at a total of twelve different growth conditions. The media were AB minimal medium [15] with 1 µg/ml of thiamine supplemented with either 0,4% acetate and 25 µg/ml uridine (Acetate medium), 0,2% glucose and 50 µg/ml uridine (Glucose medium), or 0,2% glucose, 0,5% casaminoacids and 50 µg/ml uridine (Glucose-CAA medium), and LuriaBroth medium supplemented with 0,2% glucose and 50 µg/ml uridine (LB-G medium). Three independent parallels were performed for each medium and temperature condition.

To ensure balanced growth cells were grown for 4–5 generations before sampling. At OD_450_ (or OD_600_ for LB-G medium) = 0.15, steady-state exponentially growing cells were harvested or treated with 300 µg/ml of rifampicin (Fluka) and 10 µg/ml of cephalexin (Eli Lilly) for four to five generations. Samples were harvested after the drug treatment. Both exponentially growing cells and cells treated with rifampicin and cephalexin were resuspended in TE buffer (20 mM Tris-HCl pH 7.5, 1 mM EDTA) and fixed in 70% ethanol.

### Flow cytometry

Cells fixed in ethanol were washed in 0.1 M phosphate buffer (PB) pH 9.0, and stained overnight (4°C) in the same buffer with 3 µg/ml of fluorescein isothiocyanate (FITC). The cells were washed in Tris-buffered saline (TBS) (20 mM Tris-HCl pH 7.5, 130 mM NaCl), and stained in the same buffer containing 1.5 µg/ml of Hoechst 33258 (Sigma) for at least 30 minutes. An ethanol-fixed sample of cells which contained 1, 2, 4 and 8 chromosomes was used as an internal standard. The standard was included with every sample during incubation with the Hoechst 33258 stain and was not stained with FITC.

Flow cytometry analysis was then performed using a LSRII (Becton Dickinson) equipped with an 488 nm Argon ion laser and a 355 nm Krypton laser (both Spectra Physics). The FITC fluorescence was collected through a 530/30 nm bandpass filter. A 450/50 nm bandpass filter was used to collect Hoechst 33258 fluorescence. The peaks of the FITC-negative standard were positioned at fixed channels by tuning the PMT voltage, allowing the DNA content of the FITC-positive sample cells to be accurately calculated. The data obtained from the flow cytometry measurement was processed by WinMDI (©Joseph Trotter) or FlowJo (©Tree Star, Inc) software.

### *Q*-PCR

To obtain *oriC*/*ter* ratio using quantitative PCR chromosomal DNA was purified from exponential (not drug-treated) cultures. 4–5 ml fixed culture was collected by centrifugation and lysed with 1.2% SDS and 4 mM EDTA, at 65°C for 5 minutes. The DNA was precipitated with 0.7×volume isopropanol and washed with 70% ethanol. The DNA was treated with RNaseA (Sigma-Aldrich) and proteinase K (Sigma-Aldrich) for 45 min and 1 hour respectively and then the proteins were precipitated with Protein precipitation solution (Promega). The DNA was then precipitated with isopropanol and collected by centrifugation. 5–10 ng was used in the PCR amplification (7900HT Fast Real-Time PCR system, Applied Biosystems). The primers used were 5′ GAGAATATGGCGTACCAGCA and 5′ AAGACGCAGGTATTTCGCTT for amplification of the *oriC* region and 5′ TCCTCGCTGTTTGTCATCTT and 5′ GGTCTTGCTCGAATCCCTT for amplification of the *ter* region. The fluorescent probes were 5′ Fam - 3′ Tamra with the sequence 5′- CAACCTGACTTCGGTCCGCG and 5′-CATCAGCACCCACGCAGCAA for *oriC* and *ter* respectively. The data from the samples were normalized to the data obtained from MG1655 wild type cells treated with rifampicin and cephalexin where the *oriC*/*ter* ratio is 1∶1. In an exponentially growing culture the frequency of origin to terminus copies are given by 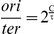, and the C phase durations were calculated by 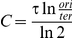. The Q-PCR analyses were carried out at least four times for each independent parallel from the selected growth conditions.

### Mathematical basis and the simulation program

**Figure 1:**
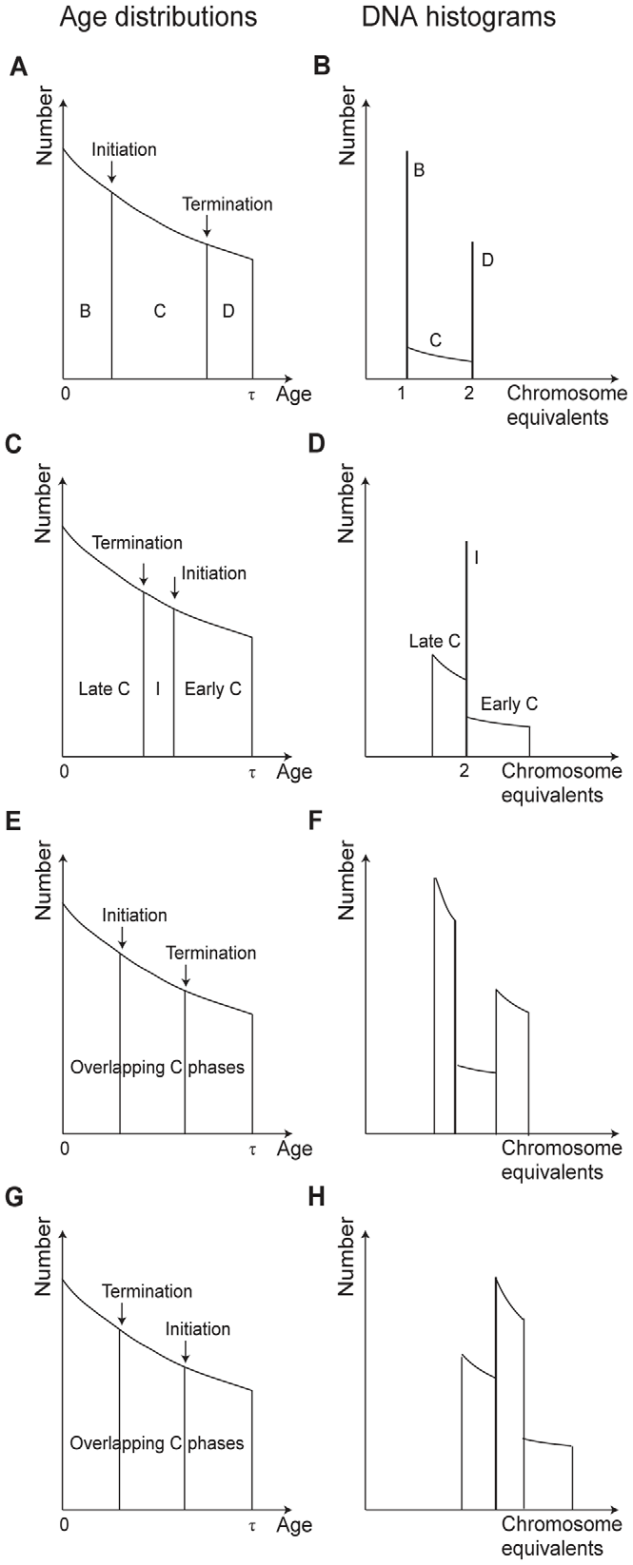
Age distributions and DNA histograms for theoretical cell populations. (A) Illustration of the age distribution (number of cells as a function of cell age) for an exponentially growing cell population. The age spans from newborn, 0, to dividing cell, τ. Initiation and termination of DNA replication occurs at the indicated ages. (B) The corresponding DNA histogram will contain a lower number of cells per DNA interval for “older” cells. (C) The age distribution of cells with initiation of replication in the previous (“mother”) generation. For these cells C phase will continue through division, from Early C to Late C. (D) In the corresponding DNA histogram cells from the period between termination and initiation (I) will accumulate in a two chromosome peak. Cells from Early C have a higher total replication rate than cells in Late C and hence less cells per DNA interval. (E and G) Equivalent age distributions for two theoretical cell populations initiating replication in the “mother” and “grandmother” generation respectively. (F and H) Since the C phases overlap in both cases, no distinct peaks will accumulate in any of the DNA histograms. Due to different total replication rates, the numbers of cells per DNA interval will vary.

In a population growing exponentially with time, which will contain cells of age t ranging from newborn (t = 0) to dividing (t = τ), the number of cells per age will decrease with age (1). The probability distribution of cells as a function of cell age is here denoted p(t), and k and n_0_ are constants.
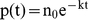
(1)At any given time there are twice as many newborn cells as dividing cells. Setting this as condition and normalizing to one gives the age distribution (2). The age of dividing cells is called τ, and can be measured in mass doubling time.
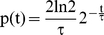
(2)Integration of the age distribution between the two ages t_1_ and t_2_ provides the portion of cells in this time interval (3).
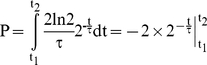
(3)Flow cytometry of a cell sample stained with Hoechst 33258 produces a histogram where the number of cells per DNA content is plotted. Cells which do not replicate their DNA (e.g. in B or D phase) contain a constant amount of DNA in this time period, and the total fraction of the population in this peak can be calculated by equation (3). The integration limits t_1_ and t_2_ are then given by the start- and stop-age of this phase. An illustration of the age distribution and the DNA histogram for a slowly growing population are illustrated in figure 1 A and B. For cells with ongoing DNA replication increasing amounts of DNA will be associated with increasing cell age, and hence decreasing number of cells per DNA content (see C-phase cells in figure 1 B). The portion of cells for each DNA channel in a histogram can still be calculated using the age distribution (3), but now for more limited time intervals.

A property of some bacteria is their ability to grow with overlapping replication cycles, so the sum of the durations of the C and D period exceeds the doubling time τ. Replicating cells of different ages can therefore have different total replication rate according to the number of replication forks at work. In a DNA histogram the parts of the population with low total replication rate will yield a higher number of cells per DNA content. The age distribution and DNA histogram of a theoretical population where cells initiate replication at two origins in the middle of the cells' life are shown as an example in figure 1 C and D. In the first part of C phase, named Early C, the cells harbour four replication forks. After cell division, in Late C, the cells harbour half that number, two forks, and thus a lower total replication rate, which will lead to more cells per DNA content.

The time it takes a cell to replicate a certain amount of DNA is inversely proportional to the total replication rate, so in a DNA histogram the fall in number of cells per DNA content will be steeper for lower total replication rate. In figure 1 D this can be seen as a steeper decrease in the number of cells per DNA content in Late C than in Early C.

An example of a cell population where initiation of replication occurs at two origins early in the cells life (instead of in the middle as shown in figure 1 C and D), is shown in figure 1 E and F. Here initiation occurs (and generates four forks) before the two old forks have terminated, and the cells in the intermediate period will then contain six replication forks. The DNA histogram has a drop in the number of cells per DNA content for the cells in the intermediate period because the rate of DNA synthesis is higher than in the periods before (where cells have two forks) and after (where cells have four forks) (figure 1 F).

Cells can also initiate replication at four origins in the “grandmother” generation. Alterations in the replication pattern (the age and generation of replication initiation and termination events) can cause the resulting DNA histogram to change quite a lot. In general, an earlier initiation age and shortened C phase will lead to a longer D phase and an increased DNA content in the population. In figure 1 G and H an example of the age distribution and corresponding DNA histogram of one population where replication initiates in the grandmother generation are shown. Here termination occurs in the current generation and at a lower cell age than initiation (C>τ, D<τ). The total replication rate will however vary. A general rule for DNA histograms is that when followed from left to right, there is a drop at the DNA content for which initiation occurs and a rise for termination. For bacteria with overlapping replication cycles the DNA content of the cells at different ages can be deduced by considering how far replication of the chromosome(s) has come relative to the total length of the C period.

Our simulation program is written in Visual Basic, so all input parameters and the resulting graphs can be handled in the corresponding Excel worksheet. The software is attached as file S1. A detailed Step-by-step guide for use of the program is included (file S2). For different choices of doubling time, chromosome size, Mb/channel and initiation generation the parameters initiation age and C phase duration can be iterated to produce theoretical DNA histograms. The theoretical histogram that is nearest in shape and position to the experimental one gives the best estimate of the parameters. For 256 DNA content intervals the numbers of cells per interval are calculated according to the age distribution (3) and plotted by the routine. A normal distribution with an adjustable standard deviation can be applied to the theoretical histograms to account for biological cell-to-cell variability in the populations. The standard deviation will also depend on the quality of the experimental histogram (applied voltage for excitation of the fluorochrome, calibration, run speed, etc). The experimental and theoretical DNA histograms are displayed in a single graph window and the deviation s (4) between them is calculated. Here x_i_ and y_i_ are the theoretical and experimental numbers of cells respectively, for the i'th DNA interval.
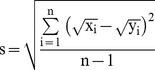
(4)To independently find the initiation age and the extent of overlap in replication cycles, the cells can be treated with rifampicin and cephalexin before harvesting to find the number of origins in the cells in the population [16]. These drugs inhibit new initiations and cell division respectively, but allow completion of ongoing replication forks. Cells will end up with 2^n^ or 2*2^n^ chromosomes (which equals origins), where n ranges from 0 to 3 and depends on the initiation generation. Initiation in the current generation gives n = 0, in the previous (mother) generation n = 1, and so on. The cells in the 2^n^ peak have not yet initiated replication and this portion can then be inserted in equation (3) to find the initiation age (setting t_1_ to zero gives this age as t_2_). In the Excel Worksheet we provide an additional tool to perform this calculation.

The doubling times of the cultures are known from the OD-measurements. As well as the initiation generation and a preliminary initiation age (described above), the DNA histogram for the rifampicin/cephalexin treated cells also provides a means to calibrate the DNA axis. The input parameter Mb/channel can be found by dividing 2^n^ times the chromosome size by the corresponding DNA content channel of the peak.

## Results

### Matching simulated and experimental E. coli DNA histograms

**Figure 2:**
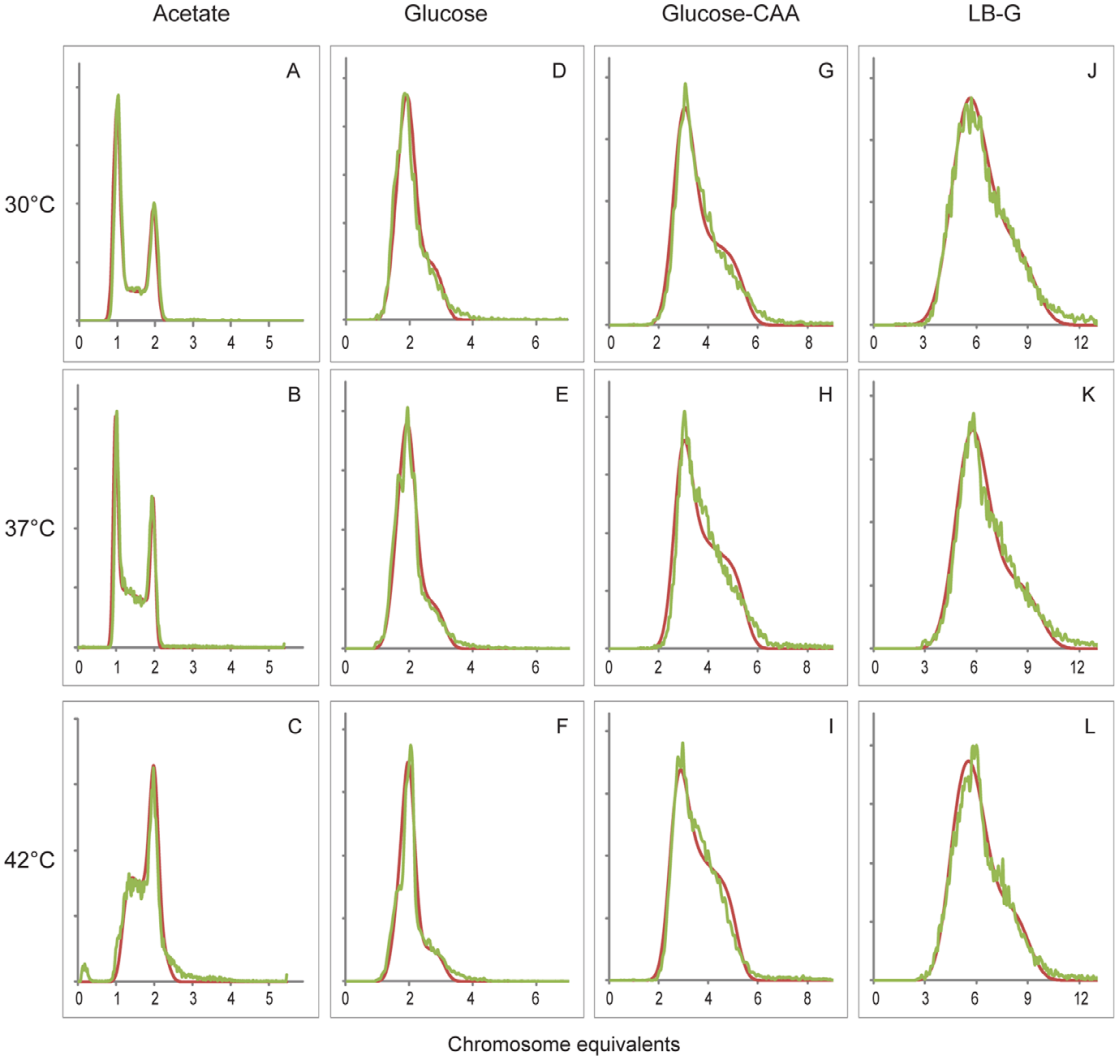
Simulations of DNA histograms to determine cell cycle parameters for *Escherichia coli*. *E. coli* cultures were grown in four different media at three different temperatures, and samples were subjected to flow cytometry to obtain the experimental DNA distributions (green curves). By iteration of cell cycle parameters in our computer program best fit theoretical DNA histograms were found (red curves). One parallel of three is shown for each temperature for Acetate medium (A–C), Glucose medium (D–F), Glucose-CAA medium (G–I) and LB-G medium (J–L). The x-axes denote chromosome equivalents, and the scale is different from medium to medium.

*Escherichia coli* strain MG1655 was grown in four different media (Acetate, Glucose, Glucose-CAA and LB-G) at three different temperatures (30°C, 37°C and 42°C). Three independent parallels were performed for each of the twelve growth conditions. Cell samples were fixed and suspended in Hoechst 33258 for staining of DNA before they were subjected to flow cytometry. The samples were also stained with FITC (see Methods). Simulations of the populations' DNA histograms were performed with iteration of cell cycle parameters until the best fit to the experimental exponential DNA histogram were obtained. A normal distribution was included in the simulation software to account for measurement uncertainty and biological variability in the culture (see Methods section). This standard deviation was also iterated and found to range from around two in the slowly growing cells with little DNA up to eight in the cultures with highest DNA content. Figure 2 displays the resulting histograms for one parallel of each growth condition. The deviations between the theoretical and experimental histograms were found to be between 1 and 3.

Note that the scale of the x-axes, denoting chromosome equivalents, vary with media for the histograms (figure 2). Cells grown in Acetate medium at 30°C or 37°C contained from one to two chromosomes, which means that the cells grow without overlapping rounds of replication. But at 42°C cells contained from above one to above two chromosome equivalents, indicating that initiation had occurred before cell division. For the other three more nutrient-rich media the shape and DNA content of the histograms seemed pretty independent of temperature. The DNA content of the populations increased from cells grown in Acetate, to cells grown in Glucose, to Glucose-CAA, to LB-G medium. In LB-G medium cells contained roughly between four and eight chromosome equivalents.

**Figure 3:**
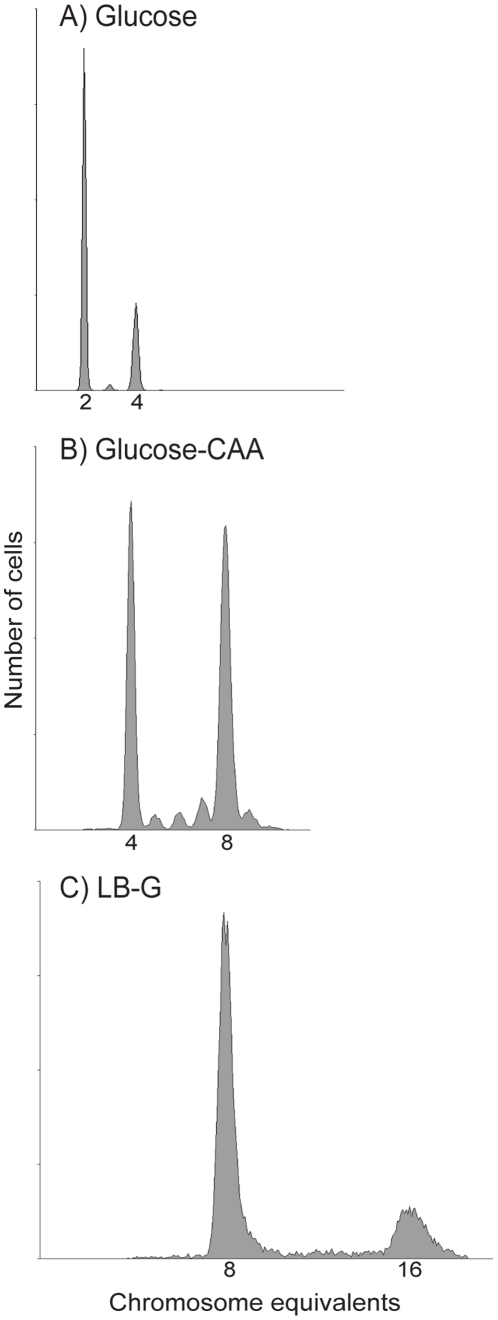
Initiation synchrony in different media. Histograms obtained by flow cytometry of rifampicin and cephalexin treated *E. coli* cells grown in Glucose medium (A), Glucose-CAA medium (B) and LB-G medium (C) at 37°C. The presence of 2 and 4 origins (A), 4 and 8 origins (B), and 8 and 16 origins (C) in the cell cultures when the drugs were added indicate that the cells initiate in the mother (A), grandmother (B) and great-grandmother (C) generation respectively. *E. coli* grown in Glucose medium and LB-G medium can be seen to initiate synchronously, but the Glucose-CAA grown cells contain subpopulations with 5, 6, 7 and 9 origins (in addition to the 4 and 8 peaks) and do apparently not fire all origins at the exact same timepoint.

Even though simulations can be made solely based on the histograms of exponentially growing cells, histograms of cells treated with rifampicin and cephalexin prior to fixation can provide an aid in determining the initiation age and generation of a cell population for cells growing with overlapping rounds of replication. DNA histograms for one parallel of such cells grown in the three richest media at 37°C are shown in figure 3. Cells grown in Glucose-CAA medium could be seen to contain other peaks in between the 4 and 8 chromosome peak (2^n^ and 2*2^n^, with n = 2), the average amount of cells in these peaks were about 10% for all three temperatures. In comparison the Glucose populations contained on average 2% such cells. This can explain the deviation in figure 2 between the right-hand side of the experimental and simulated DNA histograms for the Glucose-CAA cells (figure 2 G–I). The simulation program assumes initiation synchrony of the origins, and will hence produce histograms with a steeper decrease than that of the slightly asynchronous experimental histograms. The initiation window for the Glucose-CAA populations were calculated to over 2 minutes for the 37°C and 42°C conditions, and over 4 minutes for the more slowly growing 30°C cultures, by using the percentage of asynchronous peaks in equation (3). In the simulations of LB-G grown cells, the initiation ages were set a bit lower than the values obtained with the drug-treated cells, in order to match the shape of the exponential histograms. Notably, no asynchrony was observed for these populations, which fire at eight origins simultaneously.

### Growth of E. coli under different conditions

**Figure 4:**
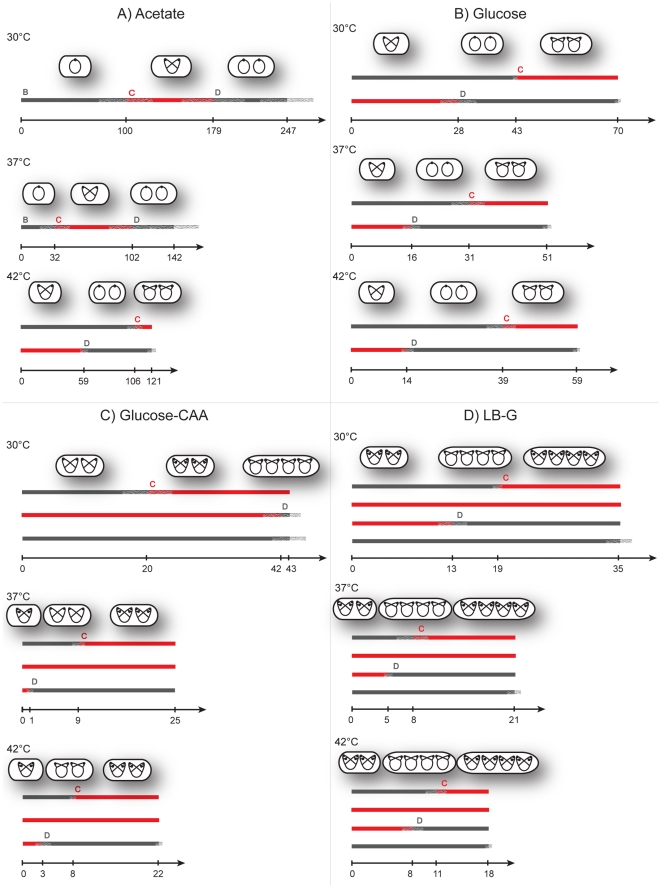
Replication patterns for *E. coli* determined by computer simulations. The average estimated C period, D period and doubling time τ for each growth condition are shown here. The arrows denote time axes, and the parallel thick lines above generations. Each line above a time axis represents one generation, where the B and D periods are shown as grey parts and the C periods as red. Plus/minus one standard deviation is indicated as grey semitransparent lines for the initiation timepoint, termination timepoint and division timepoint. The average cell ages for these events are indicated on the time axes. Displayed above these replication patterns are illustrations of cells' chromosome configurations through the cell cycle. The black dots represent origins. The patterns represent Acetate medium (panel A), Glucose medium (panel B), Glucose-CAA medium (panel C) and LB-G medium (panel D). The time axes are normalised to the same length (not to time scale) for the 30°C patterns for the various media, but are to scale within each media (in the four panels).

For each simulation the cell cycle parameters C and D that gave the best fit in the computer program were registered. Phase durations are given as output in yellow Excel cells in the simulation software (file S1). Since three parallels (the simulations of only one are shown in figure 2) were performed for each growth condition average initiation time points, termination time points and division time points with standard deviations could be calculated. In figure 4 the resulting replication patterns are shown. For populations of cells with overlapping replication rounds, some periods can span more than one generation. The average age for initiation and termination of replication and division are indicated on the time axis, and plus/minus one standard deviation for these transitions are drawn as a semitransparent grey on the generation lines in the patterns. These deviations represent the reproducibility of cultures grown at different times, and seem largest for the slowly growing Acetate cells.

Cultures grown in Glucose medium (panel B in figure 4) can be recognized as similar in replication pattern to the example in figure 1C where cells initiate at two origins in the “mother” generation. The corresponding theoretical DNA histogram illustrated in figure 1D will appear as the DNA histograms for the Glucose cells when a normal distribution is applied (Figure 2 D–F). Likewise, the patterns for Glucose-CAA cells grown at 37°C and 42°C (panel C in figure 4) are the same as for the theoretical population in figure 1G. The variability in the culture is visualized when comparing figure 1H and 2H–I.

Keeping in mind that the time axes are varied between different media, the doubling time can be seen to decrease with higher temperatures in most cases (figure 4). Even though a comparison between doubling time and DNA content of the cultures (figure 2) indicates a negative correlation for the different media, roughly the same DNA histogram can be seen for each medium at various temperatures. This is in spite of the fact that varying the temperature can cause large changes in the culture doubling times. For example all cells grown in LB-G medium, with varying doubling times according to temperature, produces the same pattern. (Since all Glucose-CAA grown cells terminate replication around cell division, before division at 30°C or after at 37°C and 42°C, these patterns are also considered highly similar.) It is interesting to note that the C-period never spanned more than two generations even in LB-G medium. This means that on each chromosome there was a maximum of six replication forks. The inter-media differences can be pronounced even though the doubling times are similar, for instance the LB-G 37°C cells and the Glucose-CAA 42°C cells have almost identical doubling times, yet dissimilar DNA histograms and consequently different replication patterns.

The cell cycle parameters obtained are summarised in table 1. The trend discussed in the previous paragraph can here be seen in the form of relatively temperature insensitive ratios of C and D phase durations to doubling time for the three richest media. Cells grown in Glucose-CAA medium at temperatures between 14°C and 37°C have also earlier been shown to display this relatively temperature independent C/τ ratio [17]. For Acetate medium it seems that the C phase duration is relatively constant for the different temperatures, even to the extent that the low doubling time at 42°C causes initiation to occur in the previous generation. Though the Acetate cells' doubling time and phase durations can vary greatly between growth parallels, the standard deviation of the C/τ and D/τ ratios is low.

**Table 1:** Cell cycle parameters for *E.coli*.

		OD	Simulation program				Q-PCR
Medium	Temperature (°C)	Doublingtime τ ±sd (min)	C period ±sd (min)	D period ±sd (min)	C/τ	D/τ	C period ±sd (min)
Acetate	30	247±25,8	79±6,7	68±5,7	0,3±0,02	0,3±0,05	-
	37	142±25,1	70±14,6	39±5,5	0,5±0,04	0,3±0,05	-
	42	121±6,1	73±10,4	61±3,1	0,6±0,07	0,5±0,02	-
Glucose	30	70±1,2	55±4,0	42±6,7	0,8±0,07	0,6±0,09	-
	37	51±2,1	36±1,5	36±0,6	0,7±0,04	0,7±0,04	41±6,9
	42	59±1,2	34±1,5	46±0,6	0,6±0,04	0,8±0,02	-
Glucose-CAA	30	43±3,1	64±1,5	44±3,1	1,5±0,07	1,0±0,00	-
	37	25±0,0	43±1,5	24±0,6	1,7±0,06	0,9±0,02	46±1,3
	42	22±0,6	39±3,2	19±1,2	1,8±0,10	0,9±0,07	43±1,7
LB-G	30	35±1,7	64±2,1	57±5,3	1,8±0,07	1,6±0,08	66±3,1
	37	21±1,2	38±2,1	36±2,6	1,9±0,08	1,7±0,04	40±2,1
	42	18±0,6	32±0,6	28±2,1	1,8±0,08	1,6±0,09	33±0,9

To obtain independent measurements of the C phase durations, we performed Q-PCR of origin and terminus markers on DNA from the following conditions: LB-G medium at all temperatures, Glucose-CAA medium at 37°C and 42°C, and Glucose medium at 37°C. The resulting C phase durations were consistent with the C periods found by simulation (table 1).

### DNA chain elongation rate during growth in acetate medium

**Figure 5:**
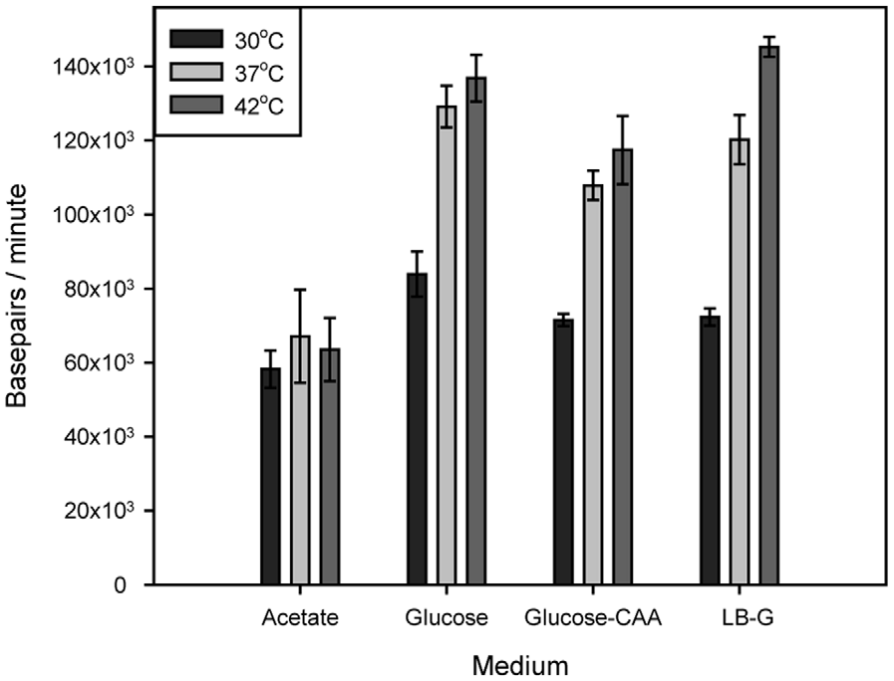
The average DNA chain elongation rate increases with temperature in all media except acetate medium. C period durations (table 1) were converted to average chain elongation rates and plotted as average number of base pairs per minute.

In general, when temperature is increased, biochemical processes speed up. We find here that this is true also for DNA replication during growth in three of the media tested (figure 5). The average DNA chain elongation rate increased from about 80 kb per minute at 30°C to around 120 kb per minute at 37°C during growth in Glucose, Glucose-CAA and LB-G medium. The growth rates (including the rates of other relevant processes) also increased with temperature and thus the replication patterns remained relatively invariable (figure 4). In acetate medium, however, the chain elongation rate did not increase with temperature. The growth rate did increase with temperature and the biochemical processes of the D period also seemed to increase in rate yielding shorter (or at least varying) D periods with higher temperature. The resulting replication patterns thus changed considerably with temperature during growth in acetate medium.

The reason for the low chain elongation rates during growth in acetate medium at high temperature is not known but could have to do with the carbon source itself. The carbon flux through the three-carbon part of the glycolysis/gluconeogenesis pathway will be in the opposite direction during growth on acetate compared to glucose [18]. In *Bacillus subtilis* indications have been found that carbon metabolism affects the regulation of replication enzymes directly [19]. It is thus possible that the nature of the carbon flux during growth on acetate affects the DNA synthesis directly, imposing a limitation on the chain elongation rate. In *B. subtilis* this regulation seems to target the replisome directly [19]. Whether this is the case in *E. coli* remains to be investigated.

## Discussion

### A simulation program for determination of cell cycle parameters for one-chromosome bacteria

We have developed a program for simulations of DNA distributions for theoretical cell cultures, and used it to compute histograms for *Escherichia coli* growing at various temperatures and with different nutrients. By adjusting cell cycle parameters, good fits to the experimental histograms were achieved for all growth conditions. This program can be used for simulations of other one-chromosome bacteria as well, as long as the genome size is known, and the cells grow as single cells. The chromosome can be both circular and linear, assuming bidirectional replication of two chromosome arms of about the same length and at about the same speed.

Earlier methods used for estimating cell cycle parameters include residual DNA replication or division during chloramphenicol exposure or thymine starvation, decay of incorporated ^125^I in DNA, membrane elution and others [3], [6], [20]. More recently marker frequency analyses determining origin to terminus ratios are commonly performed, either by Southern hybridization or Q-PCR [21], [22]. This ratio will however only produce the duration of the C phase, and flow cytometry is often carried out anyway to determine the C+D duration from the rifampicin and cephalexin treated cells. For slowly growing cells the origin to terminus ratio will approach one, and the C phase calculation becomes very uncertain. Flow cytometry based simulations are a rapid and accurate method to estimate cell cycle parameters for these populations, and have here been proven valuable for cells cultivated at higher growth rates as well.

### Largely temperature independent replication patterns of E. coli in rich media

As mentioned there are many studies on bacteria concerned with investigations of replication and segregation, especially by the aid of immunofluorescence or live-cell imaging [23], [24]. In rapidly growing cells origins, replication forks or termini are often observed as co-localized [25], [26], [27], [28]. A means to determine the cells' replication patterns is therefore required for knowledge about the exact numbers, and the variances at different cell ages, of these and other structures commonly visualized.

Our investigations of cell cycle parameters for *E. coli* grown at different growth rates revealed relatively temperature independent replication patterns in each of the three media displaying multi-folk replication. Thus, a lowered temperature, with corresponding prolonged doubling time, will not necessarily lead to fewer origin copies or replication forks. To achieve this, a change of media is often required, and should then always be accompanied by determination of the cell cycle parameters to correctly calculate the number of origins, replication forks and termini.

Although the average replication fork velocity was found to increase with temperature in three of the four media used, it was not the case in acetate medium. This demonstrates that this condition has different regulation. We suggest that each growth condition is governed by specific regulation, and therefore each condition needs to be studied separately.

## Supporting Information

File S1 **Simulation software for one-chromosome bacteria.**
(XLSM)

File S2 **Step-by-step guide for the simulation software.**
(PDF)
