# Supplementary material for: An Easy-To-Use Simulation Program Demonstrates Variations in Bacterial Cell Cycle Parameters Depending on Medium and Temperature
Source: PLoS One. 2012 Feb 13;7(2):e30981. doi: 10.1371/journal.pone.0030981 (PMC3278402; doi:10.1371/journal.pone.0030981)
Supplement: File S2 — Step-by-step guide for the simulation software. (PDF) [file pone.0030981.s002.pdf]

# Step by step guide for cells without overlapping replication cycles

If you have grown the cultures before, under the same conditions, and they are slowly growing without overlapping replication rounds, the internal standard and the FITC staining are not necessary for the cell cycle analyses. Treating cells which have only one round of ongoing replication with rifampicin and cephalexin to determine the initiation age should be avoided, since rifampicin resistant initiations can occur for slowly growing populations (Flatten *et al.*, 2009). Instead, the initiation age should be fitted directly by iteration until the theoretical and experimental histograms fit.

## Cell growth and flow cytometry

Grow cells in desired medium. Perform flow cytometry analyses (see methods section).

## Simulation program input

Note that an example experimental histogram is provided in the Excel file (green curve) for testing. See guide for cells with overlapping replication cycles for more information.

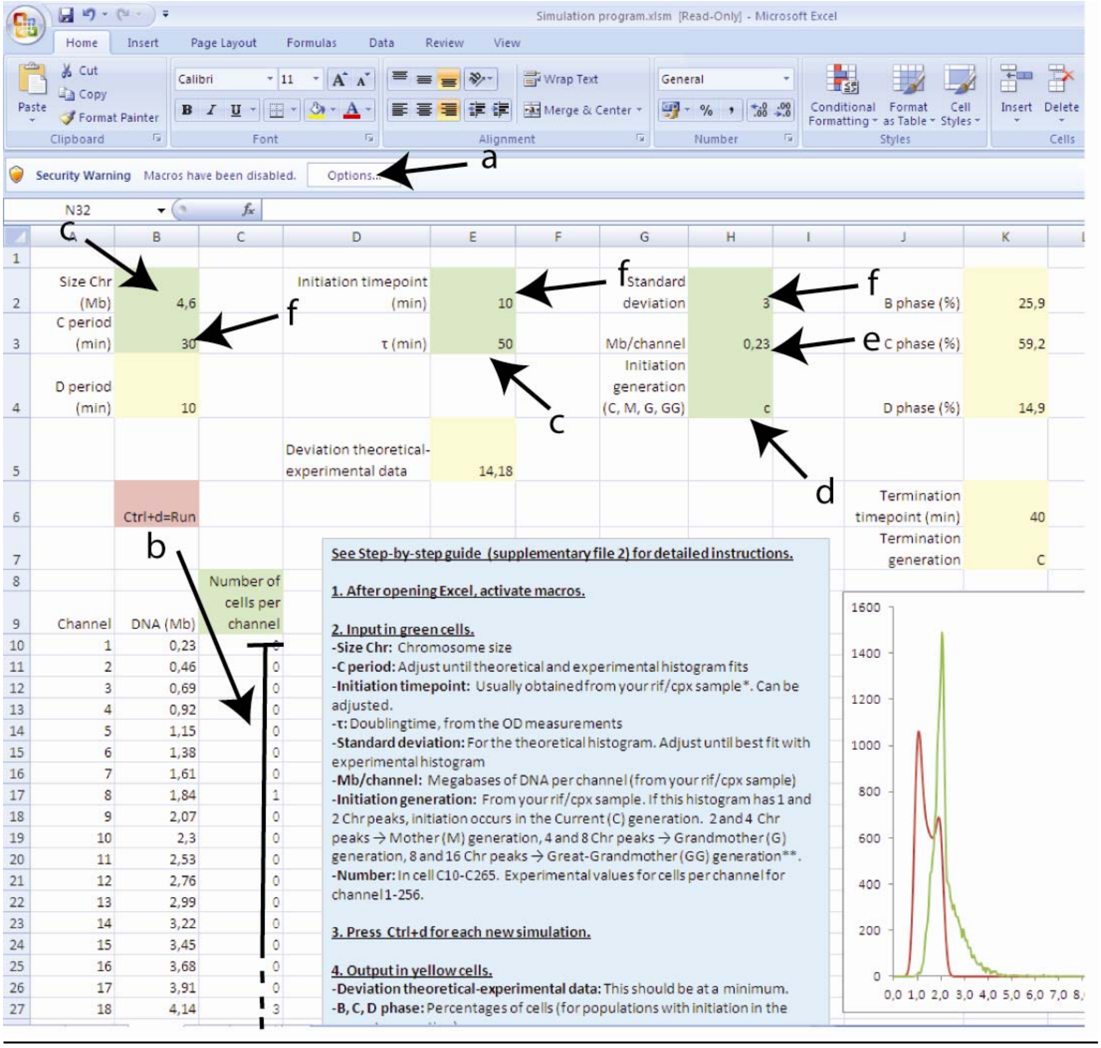

a) Open the Excel file “Simulation program” (supporting file 1), and activate macros.

  - To activate macros in Excel 10: Click the **File** tab, click **Options**. Click **Trust Center**, then **Trust Center Settings** button. Select **Macro Settings**, and **Enable all macros.** Close and reopen Excel. (Be aware that this setting is not recommended; consider disabling macros again after simulating.)
  - To activate macros in Excel 07: Press the **Options**-button in the **Security warning** banner, and select **Enable this content** in the pop-up window. If you don’t get the Security warning, click the **Microsoft Office Button**, and then click **Excel Options**. Click **Trust Center**, click **Trust Center Settings**, and then click **Macro Settings**. Select **Disable all macros with notification.** Close and reopen Excel.
  - To activate macros in Excel 03 and older: Click the **Tools** meny, **Macros**, click **Security**. Select **Medium**.

b) Insert number of cells per DNA channel of your experimental DNA histogram in Excel cells C10-C265. You should now see the green curve change to your DNA histogram. The number of cells per channel for the DNA histograms can be extracted with a Flow cytometry analysis program, f. ex. FlowJo (©Tree Star, Inc) or WinMDI (©Joseph Trotter). If the default scale is 1024 channels, convert to 256 channels before copying the number of cells per channel. See Tip 1 for instructions on how to export cells per channel from the freeware WinMDI.

c) Insert the chromosome size in cell B2, and the doubling time of the population ($\tau$) in cell E3. The doubling time can be calculated from the OD-measurements.

d) The initiation generation is the current (type C in cell H4).

e) Mb/channel (cell H3) can be found by dividing the DNA content of a peak by the peak’s DNA channel. F. ex for *E.coli,* which has a chromosome size of 4.64 Mb, a sample with the one chromosome peak in channel 40 will have: 4,64Mb / 40channels = 0,116 Mb/channel.

f) By varying the C period (cell B3) and the initiation age (E2) you can now find the best fit to your experimental sample. The standard deviation of the theoretical histogram (the width of the peaks) can be varied in cell H2. Press Ctrl+d to run the VBA macro after adjusting parameters. Compare the shape of the experimental (green) and theoretical (red) histogram to determine the fit. The quantitative deviation between the two histograms is also calculated (cell E5).

Simulation program output

- The termination age and generation are given in cells K6 and K7 respectively.
- The D period duration in B4.
- The percentage of cells in B-, C-, and D-phase are given in cells K2-4.
- In addition to the first graph, a second graph can also be found underneath. The theoretical histogram without a normal distribution is plotted in blue, so the effect of varying the standard deviation can be seen.

# Step by step guide for cells with overlapping replication cycles

Cell growth and flow cytometry

- Grow cells in desired medium. Treat cells with rifampicin and cephalexin. See methods section.
- Grow standard cells. Use a minimal medium to ensure a population containing only cells with one or two chromosomes. (See Tip 2 below.)
- Perform flow cytometry analyses as described. The samples are now stained by FITC, and the internal standard is not (Tip 3). One "sample" should be FITC positive standard (added FITC negative standard as the other samples). Make sure to keep the peaks of the FITC-negative standard at fixed channels by tuning the PMT voltage (Torheim *et al.*, 2000). (See Tip 4 for exclusion of standard.)

Simulation program input

Note that an example experimental histogram (green curve) is provided in the Excel file for testing. This histogram is the same as shown for cells grown in glucose medium at 37°C in the article (Figure 2E). By inserting the parameters found for these cells (Table 1) you should find a good fit (C period, D period by changing the initiation timepoint, initiation generation..). You may have to adjust the standard deviation.

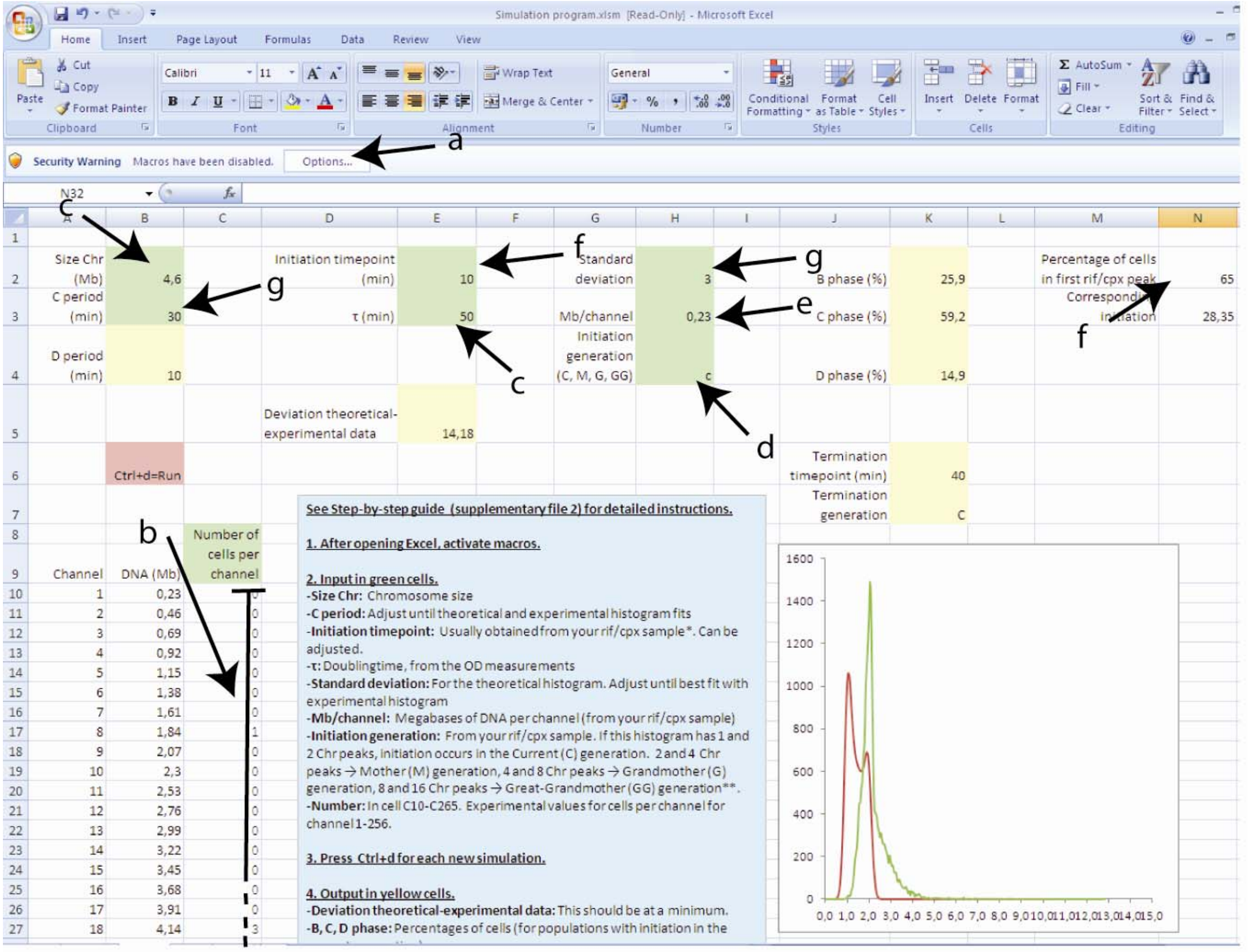


a) Open the Excel file “Simulation program” (supporting file 1), and activate macros. See guide for cells without overlapping replication cycles for details.

b) Insert number of cells per DNA channel of your experimental DNA histogram in Excel cells C10-C265. You should now see the green curve change to your DNA histogram. The number of cells per channel for the DNA histograms can be extracted with a Flow cytometry analysis program, f. ex. FlowJo (©Tree Star, Inc) or WinMDI (©Joseph Trotter). If the default scale is 1024 channels, convert to 256 channels before copying the number of cells per channel. See Tip 1 for instructions on how to export cells per channel from the freeware WinMDI.

c) Insert the chromosome size in cell B2, and the doubling time of the population (τ) in cell E3. The doubling time can be calculated from the OD-measurements.

d) To find the initiation generation (cell H4) compare the peaks in the DNA histogram of the sample treated with rif/cpx to the FITC positive standard histogram. The standard will contain cells with one or two chromosomes.

   - If the rif/cpx sample contains one and two chromosome equivalents, then the initiation occurs in the current generation (type C in cell H4). See guide for cells without overlapping replication rounds.

- If the rif/cpx sample contains two and four chromosome equivalents, then the initiation occurs in the mother (type M in cell H4).
- If the rif/cpx sample contains four and eight chromosome equivalents, then the initiation occurs in the grandmother (type G in cell H4).
- If the rif/cpx sample contains eight and sixteen chromosome equivalents, then the initiation occurs in the great grandmother (type GG in cell H4).

e) To find Mb/channel (cell H3), use the histogram of the rif/cpx treated sample. Divide the DNA content of a peak by the channel number. F. ex for *E.coli,* which has a chromosome size of 4.64 Mb, a sample containing two and four chromosomes in channel 60 and 120 will have: 2*4,64Mb / 60channels = 0,155 Mb/channel.

(If you experience some non-linearity in the fluorescence signal, the rif/cpx peaks nearest the bulk of the experimental histogram should be used.)

f) Calculation of the initiation age can be performed in the Excel sheet. Insert the percentage of the population in the first peak in the histogram of the rif/cpx treated sample in cell N2. Make sure the correct initiation generation is already inserted in cell H4. The initiation timepoint is then given in cell N3, and can be inserted in cell E2.

g) By varying the C period (cell B3) you can now find the best fit to your experimental sample. The standard deviation of the theoretical histogram can be varied in cell H2. Press Ctrl+d to run the VBA macro after adjusting parameters. Compare the shape and DNA axis placement of the experimental (green) and theoretical (red) histogram to determine the fit. The quantitative deviation between the two histograms is also calculated (cell E5). The initiation age can also be adjusted by a few minutes. (Simulations can also be performed without the use of a rif/cpx sample, see Tip 5 below.)

Simulation program output

- The termination age and generation are given in cells K6 and K7 respectively.
- The D period duration in B4.
- In addition to the first graph, a second graph can also be found underneath. The theoretical histogram without a normal distribution is plotted in blue, so the effect of varying the standard deviation can be seen.
- Not all parameter sets are valid for the software. If the box “Microsoft Visual Basic, Overflow” appears, press End, and vary the C-period by a minute.

## Tips

- Tip 1: How to extract number of cells per channel from the freeware WinMDI.

  WinMDI
  - Open the file containing the exponential histogram, edit the x-axis to 256 channels
  - Press **file/save as**
  - In **Save file format**: select **Tabbed txt**
  - Press **Save** (and choose location)

  Microsoft Word
  - Open the file saved in WinMDI
  - Press **Edit/Replace/More/Special**
  - Select ”**Find what**:”
  - Select **Special** and press ”**Tab character**”
  - Select ”**Replace with**”
  - Select **Special** and press ”**Manual line break**”
  - Press ”**Replace all**” and **Ok**. The numbers are now arranged in a column.
  - Select all (**CtrlA**) and copy (**Ctrl C**)
  - Insert the numbers in the simulation program, cells C10-C265

- Tip 2: You should ideally use a standard with DNA content in about the same range as your sample cells. So if you find that your sample cells' average DNA value is high, you may consider making a new standard containing cells with two and four or four and eight chromosomes by treating more rapidly growing cells with rifampicin and cephalexin.

- Tip 3: If estimation of the cell size by FITC staining is not of interest, the internal standard can be stained by FITC instead of the samples to save time.

- Tip 4: If you are positive that your flow cytometer is stable you can exclude the standard. Run every other exponential and rif/cpx sample and make sure the positions of the rif/cpx peaks do not shift.

- Tip 5: If you do not want to or are unable to treat your cells with rif/cpx you can iterate the initiation timepoint as well as the C period and standard deviation (g). The initiation generation will also have to be estimated (d). For the Mb/channel parameter (e), use the histogram of the FITC positive standard instead.

# References


Flatten, I., Morigen & K. Skarstad, (2009) DnaA protein interacts with RNA polymerase and partially protects it from the effect of rifampicin. *Mol Microbiol* **71**: 1018-1030.

Torheim, N. K., E. Boye, A. Lobner-Olesen, T. Stokke & K. Skarstad, (2000) The Escherichia coli SeqA protein destabilizes mutant DnaA204 protein. *Mol Microbiol* **37**: 629-638.
